# Scalds and Burns

**Published:** 1857-11

**Authors:** D. Meredith Reese


### SCALDS AND BURNS.

*D. Meredith Reese, M. D., LL.D.*

THE veteran editor of the *American Medical Gazette* of New York, has never written a more truthful and more universally useful and practical article than the following. It should be preserved in some conspicuous place in every household in the land, we only adding, that until the flour is procured, *let the injured part be put under cold water, and the pain instantaneously ceases.* Apply the dry flour.

"We still see reported, almost daily, an appalling number of deaths by burns and scalds, not one of which, we take upon ourself to say, need prove fatal, or would do so, if a few pounds of wheat flour could be promptly applied to the wounds made by fire, and repeated, until the inflammatory stage had passed. We have not known a fatal case of scalding or burning in which this practice has been pursued, during more than thirty years' experience, and have treated hundreds in both public and private practice. We have known the most extensive burns by falling into cauldrons of boiling oil, and even molten copper, and yet the patient rescued by this simple and cheap remedy, which, from its infallible success, should supplant all the fashionable nostrums, whether oil, cotton, lead-water, ice, turpentine, or pain extractors, every one of which has been tried a thousand times with fatal results, and the victims have died in excruciating agony, when a few handfuls of flour would have calmed them to sleep, and rescued them from pain and death. Humanity should prompt the profession to publish and republish the facts on this subject, which are established by the authority of standard medical works on both sides of the Atlantic."

---

### HOW A DOCTOR LOOKS.

"*Three faces wears the Doctor: when first sought*
An Angel's; and a God's, the cure half-wrought;
But when, that cure complete, he seeks his fee,
Beelzebub looks less terrible than he."

